# An Anhydrous Sodium Chloride Skin Preservation Model for Studies on Keratinocytes Grafting into the Wounds

**DOI:** 10.3390/pharmaceutics13122078

**Published:** 2021-12-04

**Authors:** Anna Domaszewska-Szostek, Magdalena Gewartowska, Marek Stanczyk, Beata Narowska, Maria Moscicka-Wesołowska, Waldemar Lech Olszewski

**Affiliations:** 1 Department of Human Epigenetics, Mossakowski Medical Research Institute, Polish Academy of Sciences, 02-106 Warsaw, Poland; 2 Electron Microscopy Research Unit, Mossakowski Medical Research Institute, Polish Academy of Sciences, 02-106 Warsaw, Poland; mgewartowska@imdik.pan.pl; 3 Faculty of Medicine, Lazarski University, 02-662 Warsaw, Poland; stanczyk@poczta.onet.pl; 4 Department of Advanced Material Technologies, Faculty of Chemistry, Wroclaw University of Science and Technology, 50-370 Wroclaw, Poland; narowska.beata@gmail.com; 5 Department of Surgical Research and Transplantology, Mossakowski Medical Research Institute, Polish Academy of Sciences, 02-106 Warsaw, Poland; maria.moscickawesolowska1968@gmail.com (M.M.-W.); waldemar.l.olszewski@gmail.com (W.L.O.)

**Keywords:** keratinocytes, epidermis, skin transplantation, anhydrous NaCl

## Abstract

Background. Human skin is needed for covering large body areas lost by trauma. The shortcomings of contemporary methods of skin storage are limited preservation time and high immunogenicity if allogeneic. Methods. We investigated whether long-lasting skin preservation in anhydrous sodium chloride (NaCl) may be the source of keratinocytes (KCs) for transplantation. Dehydrated skin fragments were preserved for a time frame from 1 week to 12 months. Then, skin fragments were rehydrated, and KCs were isolated. The viability of KCs was assessed in viability/cytotoxicity test. NaCl-preserved KCs were cultured for 7 days and transplanted to the dorsum of SCID mice. Results. The morphology of NaCl-preserved KCs was unaltered. KCs from all epidermal layers could be identified. All grafts were accepted by the recipients. Transplanted KCs: synthesized keratins 10 and 16 expressed antigens specific for stem cells and transient-amplifying cells, and remained HLA-I-positive. Moreover, they expressed the proliferative marker PCNA. Cells isolated from transplants remained viable and produced enzymes. Conclusions. Transplantation of KCs obtained from human skin and stored in anhydrous NaCl may be considered for the closure of extensive skin wounds. The originality of this method consists of an effective storage procedure and easy preparation of keratinocytes for transplantation.

## 1. Introduction

The need for human skin to cover large body areas lost by injury is fulfilled only in a small number of cases. This especially applies to regions where access to specialized health care is limited. The gold standard of care for extensive skin injuries is wound debridement, followed by skin grafting. The only solution that meets the demand for skin grafts is long-lasting tissue or cells banking [1]. The main currently applied methods of storage include: glycerol cryopreservation, vitrification, and storage in saline, RPMI, Ham, or DDEM at 4 degrees Celsius or room temperature [2]. Such transplants, if allogeneic, are rejected due to their high immunogenicity. Until the moment of rejection, they fulfill the role of a physiological dressing, incomparably more effectively than artificial skin equivalents. Large doses of immunosuppressants may prolong the graft survival until the granulating tissue underneath is covered by the recipient keratinocytes [3].

Our past research investigated the preservation of various cells and tissues in anhydrous NaCl at room temperature [4,5]. This idea arose while performing research in tropical countries, where the freezing devices for collected tissues were not easily accessible. Dehydrated tissue samples, preserved in anhydrous NaCl, and stored at room temperature, maintained an unchanged structure for months [6]. Hepatocytes preserved in anhydrous NaCl presented an almost unchanged cell morphology when rehydrated [7]. The anhydrous NaCl preservation of arteries allowed for long-term storage, spare arterial wall structure, and a decrease in antigenicity [8]. Skin is resistant to prolonged ischemia, low temperature and dehydration, showing the ability to survive for a long time in the state of metabolic reduction [4,9,10,11]. In our previous studies, we proved that osmotic dehydration maintains the histological structure of the skin and might be a method for skin graft storage [12]. It turned out that fragments of human skin stored in an anhydrous NaCl at room temperature for weeks, or even months, after desalination and rehydration, expressed specific antigens for epidermal, dermal as well as skin-resident immune cells [12]. We documented that human skin preserved in anhydrous, pulverized NaCl for months and transplanted to skin wounds of SCID mice is taken by the recipient. The grafts were characterized by the intensive proliferation of KCs seen on the immunohistochemical section stained with BrdU. Clinically, hyperkeratosis was evident [4]. Dehydration in NaCl prevented the proliferation and subsequent transplantation to SCID mice brought about restarting of mitoses [4]. Cell-based therapies for covering granulating wounds are an alternative for allogeneic skin grafts. Transplanted allogenic KCs evoke a minor immune response compared to skin grafts [13].

The first goal of the current study was to develop a method for long-term keratinocytes preservation.

The second goal was to investigate whether the KCs isolated from skin fragments preserved in anhydrous NaCl retained their morphology and functioned if transplanted to the wound. KC transplantation has the potential to improve wound healing without major surgical procedures. This is important for traumatized patients, where standard skin grafting can be a burden [14]. Transplanted KCs deliver cytokines, chemokines, and growth factors necessary to control cell proliferation and differentiation [15]. They also induce angiogenesis, innervation and modify the inflammatory process in wounds [16].

The question arises as to whether KCs isolated from skin grafts preserved in anhydrous NaCl would undergo mitosis after transplantation. To address this question, we isolated KCs from anhydrous NaCl-preserved human skin grafts. Then, KCs were transplanted to the skin wounds of SCID mice. Wound healing was followed and documented. The presence of HLA-1 molecule was used for the differentiation between human and mice cells after grafting. The expression of CK10 and CK16 was investigated to assess the newly formed epidermis. Epidermal stem cells markers: p63 (keratinocytes stem cells) and CD 29 (keratinocytes stem and transient amplifying cells), as well as PCNA (Proliferating cell nuclear antigen), were used to determine the regenerative potential of transplanted KCs. Staining for CD1a (Langerhans cells) CD4, CD8 (T lymphocytes) and CD20 (B lymphocytes) were used for the detection of human immune-competent passenger cells in the KCs-transplanted healing wound.

## 2. Materials and Methods

### 2.1. Skin Preservation in Anhydrous Sodium Chloride

Skin preservation in anhydrous NaCl was performed according to the procedure described by Olszewski et al. [6]. The skin for KCs isolation was obtained from fifty donor cadavers of Caucasian origin. Full-thickness skin samples consisting of complete epidermis and dermis, 3 × 3 cm in size each, with no signs of dermatological issues, were obtained from the femoral region of the lower limbs. Skin samples were excised manually using a scalpel with a #15 blade. Once harvested, the grafts were trimmed of all underlying adipose and hair structures. The grafts were collected in sterile conditions. Blood and tissue fluid were removed by gently squeezing the skin on the sterile gauze. Pulverized NaCl was dehydrated at a temperature of 240 °C for 1 h. Skin grafts were placed in a pulverized and dehydrated NaCl in a volume ratio of 1:5 and stored in aluminum foil for 1 week to 12 months at 4 °C. Prior to transplantation, skin was rehydrated by immersion in 0.9% NaCl with 1% of bovine serum albumin at room temperature. Rehydration was repeated 3 times for 30 min. The volume ratio of 0.9% NaCl to tissue volume was 10:1.

### 2.2. Keratinocytes Isolation

Human keratinocytes were isolated according to the protocol published by Domaszewska-Szostek et al. [17]. Human skin sections were digested for 24 h in 0.3 M trypsin solution (bovine pancreatic trypsin; activity: 10.000 units/mg, Merck) with 0.025% EDTA. The epidermis was then separated and washed in PBS w/o calcium and magnesium, with antibiotics and fungicides. Cells were separated mechanically using a strainer with a pore size 40 µm (Sigma, Steinheim, Germany). Keratinocytes were washed twice in PBS by centrifugation for 10 min at 1500 rpm.

### 2.3. Cell Viability

Cell viability was determined using the Viability/Cytotoxicity Assay Kit for Live and Dead cells (Biotium, Hayward, CA, USA) according to the manufacturer’s instructions.

### 2.4. Keratinocytes Culture

Before the transplantation, KCs isolated from preserved skins were seeded at 0.5 × 10^6^ cells/mL in flat-bottom plates (Becton Dickinson, Erembode-gem, Belgium) and were cultured in KGM^TM^ Gold Keratinocyte Growth Medium BulletKit^TM^ (Lonza) supplemented with 10 U/mL of penicillin and 10 μg/mL streptomycin (Sigma) for 7 days. Cells were cultured at 37 °C in the presence of 5% CO_2_ in the NU-4950 incubator (NuAire, Plymouth, MN, USA).

### 2.5. Animals

We used sixty male SCID mice (20 to 25 g body weight, 8 to 9 weeks old), bred and maintained in our facility at Mossakowski Medical Research Institute. Mice were maintained in autoclaved cages covered with securely fitted filter covers. They received sterilized rodent laboratory chow and water ad libitum. All experimental animals were treated in accordance with the guidelines of the ethics committee of the Polish Academy of Science. The experimental design of the study was approved by the 4th Local Ethics Committee for Experiments on Animals, Warsaw, Poland (certificate of approval 24/2009).

### 2.6. Creation of Skin Wounds

The surgical wounds were performed under general anesthesia (Ketamine + Xylazine, 50 mg/kg BW + 5 mg/kg BW). Site, size, and depth of the wounds were standardized for all experiments. Wounds were created by excision of a circle area 15 mm in diameter of the full thickness of the skin on the mice back. Wounds were covered with Parafilm M Sigma Aldrich dressing.

### 2.7. Keratinocytes Grafting

Keratinocytes suspended in 200 µL of 0.9% NaCl were injected between the Parafilm dressing and the wounds bed. For grafting, we used KCs in numbers ranging from 0.5 × 10^6^ to 14.0 × 10^6^. After the cell placement, the Parafilm dressing was secured with the gauze dressing.

### 2.8. Healing Control

The wounds were controlled on days 7, 14, 21, 30 and 45 of the study. Animals were sacrificed by cervical dislocation on each of these time endpoints; 5 mice on days 7, 14, 30 and 45; and 25 mice on day 21. Skin biopsies encompassing the entire wound with a 1–2 mm margin were harvested for histologic and enzymatic evaluation.

### 2.9. May-Grünwald-Giemsa Staining Procedure

The slides were fixed in pure methanol for 15 min, air-dried and placed in May-Grünwald stain for 5 min. Next, they were washed in Trizma Buffer (20–70 mmol/L), pH 7.2, for 1.5 min. Then, the slides were placed in Giemsa solution (diluted 1:20 with deionized water) for 15 min, rinsed in deionized water, and air-dried. All reagents were obtained from Sigma Aldrich.

### 2.10. Phenotypic Characterization of Keratinocytes

To identify KC phenotypes, cytospins were fixed in 4% paraformaldehyde in PBS for 15 min and rinsed three times with PBS without Ca^2+^ and Mg^2+^. For the identification of nuclear antigens, cells membranes were permeabilized with 1% Triton X-100 in PBS for 15 min at room temperature. Next, cytospins were incubated for 1 h with a blocking mixture of 5% albumin in PBS. The mixture was then removed and primary antibodies, anti HLA-1, CD1a, CD4, CD8, CD20 (all DakoCytomation, Glostrup, Denmark) cytokeratin 10 (M7002), cytokeratin 16 (sc-53255), PCNA (sc-25280), p63 (sc-8431) and CD29 (sc-9970), diluted 1:50 in a blocking mixture, were applied. Incubation with primary antibodies was performed overnight at 4 °C. Cytospins were washed three times for 5 min in PBS without Ca^2+^ and Mg^2+^. The secondary antibody, conjugated to fluorochrome Alexa Fluor 633 (rabbit anti-mouse IgG; Invitrogen, OR, USA), was applied for 30 min at a dilution of 1:500 and placed in the darkroom. The cell nuclei were stained with 5 μM Hoechst 33,258 dye solution in PBS for 30 min at room temperature. Then, the cytospins were washed three times for 5 min in PBS, and cells were mounted with DakoCytomation Fluorescent Mounting Medium (DakoCytomation). Skin specimens were snap-frozen in ice-cold acetone and cut on a cryostat into 20 μm thick sections and mounted onto polylysine-treated slides. The rest of the staining procedure was the same as for cytospins. The analysis was performed with a Zeiss confocal microscope LSM 510 (Carl Zeiss) equipped with helium-neon and argon lasers. The photographs were taken with the Zeiss LSM 510 v. 3.2. For counting the positive cells, Cell Imaging Software for Life Science Microscopy (Olympus, Japan) was used. Cells were counted in five different fields. This method was applied for staining of all types of KCs used in the study.

### 2.11. Metabolic Activity of KCs

Metabolic activity of KCs isolated from fresh skin and KCs isolated from skin preserved for 1 month in anhydrous NaCl was investigated immediately after KCs isolation and 21 days after grafting using API ZYM test (bioMérieux). The API ZYM system containing 19 chromogenic substrates was used to detect extracellular acid and alkaline phosphatases, aminopeptidases, proteases, esterase-lipase, phosphoamidase, and glycosidases in cells isolated from the fresh, preserved, and preserved and transplanted skin. KCs isolated from skin were homogenized; then, experiments were performed according to the manufacturer’s instructions.

### 2.12. Statistical Analysis

Statistical analysis of the experimental data was performed using a one-way ANOVA followed by Tukey’s Multiply Comparison Test for post hoc comparisons. Calculations were performed in Prism 5 (GraphPad Software, Inc., San Diego, CA, USA). All data are reported as the mean of 5 independent experiments ± SD. Statistical significance was defined as *p* < 0.05.

## 3. Results

### 3.1. Viability of KCs Isolated from Anhydrous NaCl-Preserved Skin

**Figure 1:**
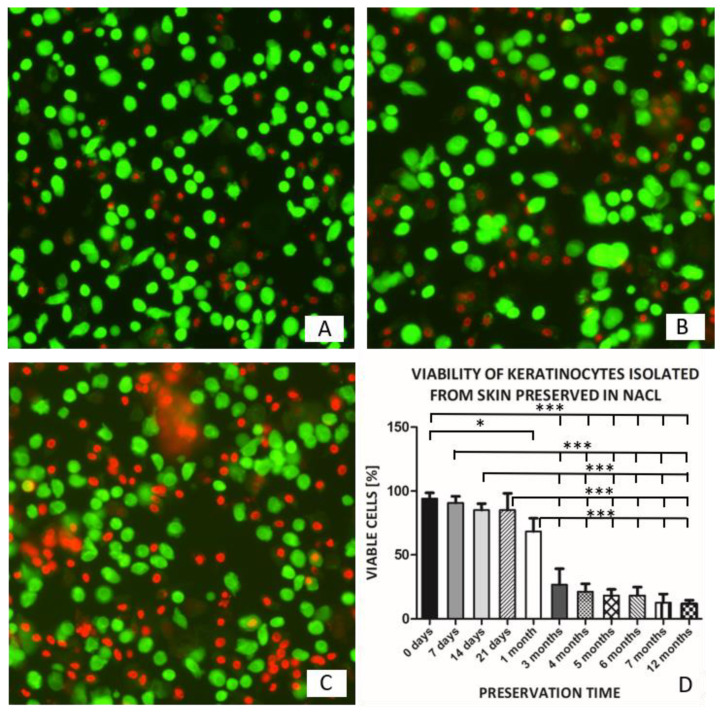
Keratinocytes isolated from skin preserved for: (**A**) 7 days, (**B**) 14 days, (**C**) 3 months. Green-stained live cells, red-stained dead cells (Viability/Cytotoxicity Assay Kit for Live and Dead cells) magnification 20×. (**D**) Viability of keratinocytes isolated from skin preserved for 0 (KCs isolated from fresh skin) to 12 months (* *p* < 0.05, *** *p* < 0.001).

The viability of KCs isolated from skin preserved in anhydrous NaCl decreases with preservation time. The viability of KCs isolated from skin preserved for 7 to 21 days decreased from 90.7% to 85% and reached 68.3% after a month. KCs isolated from skin preserved for 3 to 6 months revealed approximate viability of 20%. For the skin preserved for 7 months or more, the viability of isolated KCs decreased to 12%. Only some of the large KCs from the granular layer showed viability if the skin was preserved for 12 months (Figure 1, Table 1).

A one-way ANOVA followed by Tukey’s Multiple Comparison Test revealed a statistically significant decrease in the viability of keratinocytes obtained from skin preserved for 1 month or more, compared to non-preserved control and skin preserved for 21 days or less. (F_10,22_ = 61.91, *p* < 61.91).

These results led us to continue the experiments on KCs isolated from NaCl-preserved skin up to time limit of 6 months (18% of the viability of the isolated KCs population).

### 3.2. Morphology of KCs Isolated from Anhydrous, NaCl-Preserved Skin

**Figure 2:**
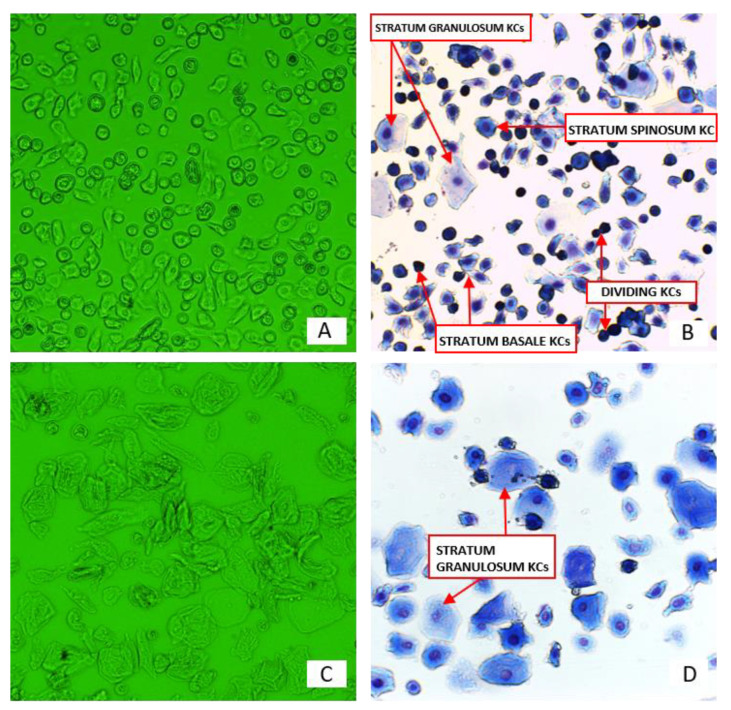
Keratinocytes isolated from skin preserved for 3 months (**A**,**B**) and from skin preserved for 6 months (**C**,**D**). (**A**) KCs culture, magnification 20×. (**B**) MGG staining, magnification 20×. (**C**) KCs culture, magnification 40×. (**D**) MGG staining, magnification 40×.

KCs isolated from skin preserved in anhydrous sodium chloride for up to 3 months maintained an unchanged morphology (Figure 2). The observation of cells in culture and May-Grunwald-Giemsa (MGG) staining revealed that keratinocytes of all layers are unaltered. Preservation in anhydrous NaCl for 6 months or more resulted in changes of morphology in keratinocytes isolated from rehydrated skin. No dividing cells from the basal layer or cells from the spinous layer were observed. The only cells that maintained an unaltered morphology were cells from the granular layer and they were more abundant than in the fraction of KCs isolated from skin preserved for 3 months. This population consisted of nucleated keratinocytes and desquamated cells.

### 3.3. Optimal Number of KCs for Grafting

Analysis to designate the optimal concentration of KCs used for grafting was performed on an experimental group of medium time of NaCl skin preservation (3 months). There are data available, which suggest that skin wound sin SCID mice after KCs grafting heal completely within 3 weeks [18]; therefore; we have chosen a follow-up of 21 days for the first set of experiments.

For grafting, we used KCs in numbers ranging from 0.5 × 10^6^ to 14.0 × 10^6^. We observed the clinical outcome on wound healing in terms of epithelialization area of the wound bed and the percentage of KCs positive for HLA-1, CK10, CK16, PCNA, p63, and CD29 in the newly formed epidermis.

**Figure 3:**
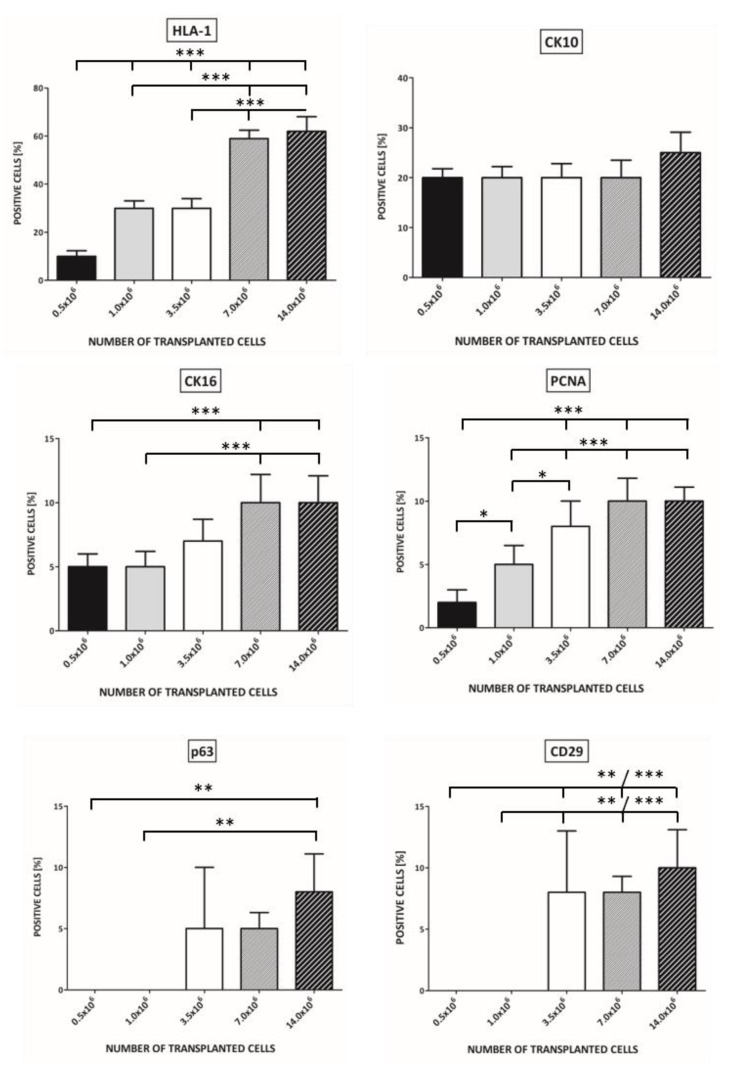
The percentage of keratinocytes in newly formed epidermis positive for HLA-1, CK10, CK16, PCNA, p63, and CD29 depending on the number of transplanted cells. The follow-up time was 21 days. Statistically significant results are shown at the bar graph (* *p* < 0.05, ** *p* < 0.01, *** *p* < 0.001).

HLA-1 expression was dependent on the number of transplanted cells. For 0.5 × 10^6^ grafted KC, only 10% of positive cells were observed in the epidermis after 21 days. For 14.0 × 10^6^ KC grafted, the number of HLA-1 positive cells was 62%. After transplantation of at least 3.5 × 10^6^ of keratinocytes, the expression of HLA-1 was detected in cells from all layers of the newly forming epidermis (F_4,20_ = 90.01, *p* < 0.0001). (Figure 3).

After the transplantation of 0.5 x10^6^ keratinocytes, the expressions of both CK10 (F_4,20_ = 2.78, *p* = 0.54) and CK16 (F_4,20_ = 12, *p* < 0.0001) were detected in granular and spinous layer of newly formed epidermis (Figure 3).

We could not identify cells positive for p63 (F_4,20_ = 8.471, *p* = 0.0004) and CD29 (F_4,20_ = 15.98, *p* < 0.0001) when grafting was less than 3.5 × 10^6^ (Figure 3).

The percentage of keratinocytes positive for PCNA was 2 ± 1.0 in the case of transplantation of 0.5 × 10^6^ KCs and rose by up to 10 ± 2.2 for 7.0 × 10^6^ and 10 ± 2.1 for 14.0 × 10^6^ grafted cells. (F_4,20_ = 25.64, *p* < 0.0001) (Figure 3).

The more cells which were transplanted, the higher the observed expression of investigated antigens. In case of 7 × 10^6^ and 14.0 × 10^6^ transplanted KCs, the expression of antigens was comparable.

The clinical outcome in terms of the epithelialization area of the wound bed stood was in agreement with the histology. There was no further significant clinical improvement in the wound healing for KCs transplanted in concentrations over 7 × 10^6^ per standard wound of 1.5 cm in diameter.

These results led us to continue grafting with KCs in a concentration of 7.0 × 10^6^ per standard wound area, as the outcome of KCs grafting in a concentration of 7.0 × 10^6^ and 14.0 × 10^6^ per standard wound area were comparable.

### 3.4. Follow-Up and Endpoint of Wound Healing

Based on the above results, we continued research on KCs isolated from skin preserved for 3 months, with 7.0 × 10^6^ KCs grafted to the area of standard skin wound. To verify the endpoint of wound healing follow-up time on our model, we observed the healing of KCs grafted wounds for the time frame from 7 days to 45 days.

**Figure 4:**
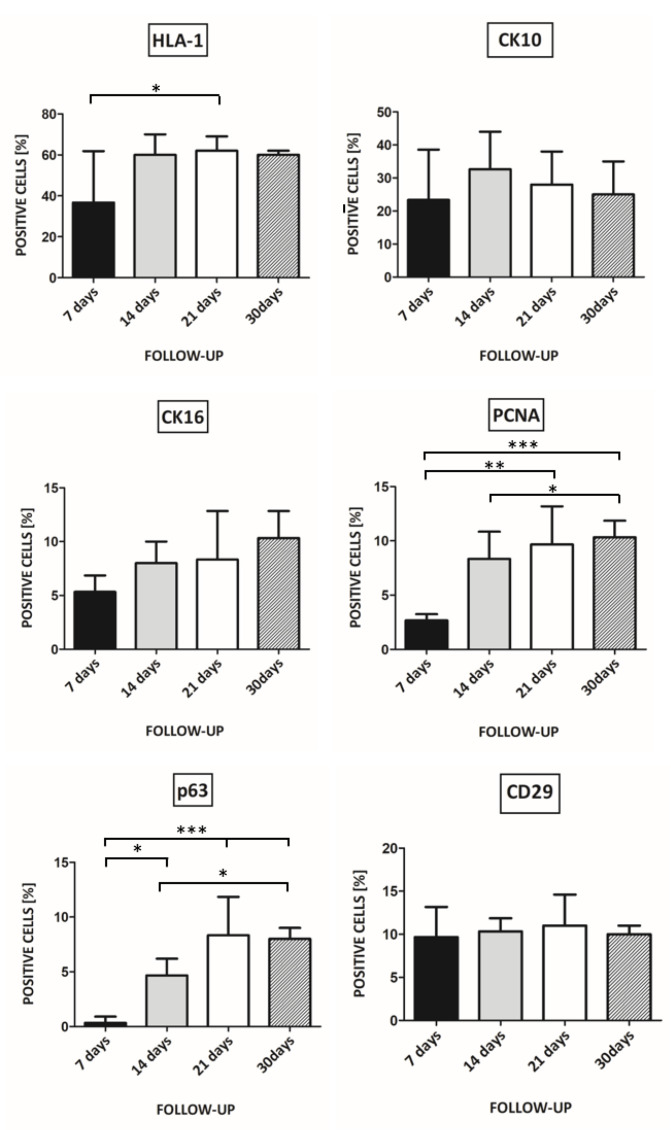
The percentage of keratinocytes in newly formed epidermis positive for HLA-1, CK10, CK16, PCNA, p63, and CD29 depending on follow-up time. Statistically significant results are shown at the bar graph (* *p* < 0.05, ** *p* < 0.01, *** *p* < 0.001).

After 7 days of transplantation, 36.7% of KCs expressed HLA-1. After more than 14 days of transplantation, the percentage of transplanted KCs expressing HLA-1 increased by approximately up to 60% (Figure 4). A one-way ANOVA followed by Tukey’s Multiple Comparison Test revealed statistically significant results (F_3,16_ = 3.700, *p* = 0.0339). With respect to HLA-1, *p* < 0.05 was obtained when comparing a follow-up time of 7 to 21 days.

There were no statistically significant changes in CK10 and CK16 expression. (Figure 4).

Regarding PCNA, statistical significance was obtained comparing 7 days of follow-up vs. 21 days (*p* < 0.01), 7 days follow-up vs. 30 days (*p* < 0.001), and 14 days of follow up vs. 30 days (*p* < 0.05) (F_3,16_ = 10.13, *p* = 0.0006) (Figure 4).

For p63 expression, statistical significance was *p* < 0.05 for the comparison of 7 days vs. 14 days and 14 days vs. 21 days. Significance *p* < 0.001 (F_3,16_ = 17.60, *p* < 0.0001) was obtained for the comparison of 7 days vs. 21 days and 7 days vs. 30 days (Figure 4).

We did not observe statistically significant changes in CD29 expression (Figure 4).

The 7 days of follow-up time turned out to be too short for the complete formation of the human epidermis. On the other hand, for the follow-up of 45 days and over, colonization of the wound with the host KCs occurred. Based on the above results, the follow-up time and endpoint for further healing study were set on 3 weeks.

According to the results obtained in all preliminary studies, we distinguished the following experimental groups (Table 2) for a further investigation of the influence of skin preservation in NaCl on the suitability of keratinocytes for transplantation.

**Figure 5:**
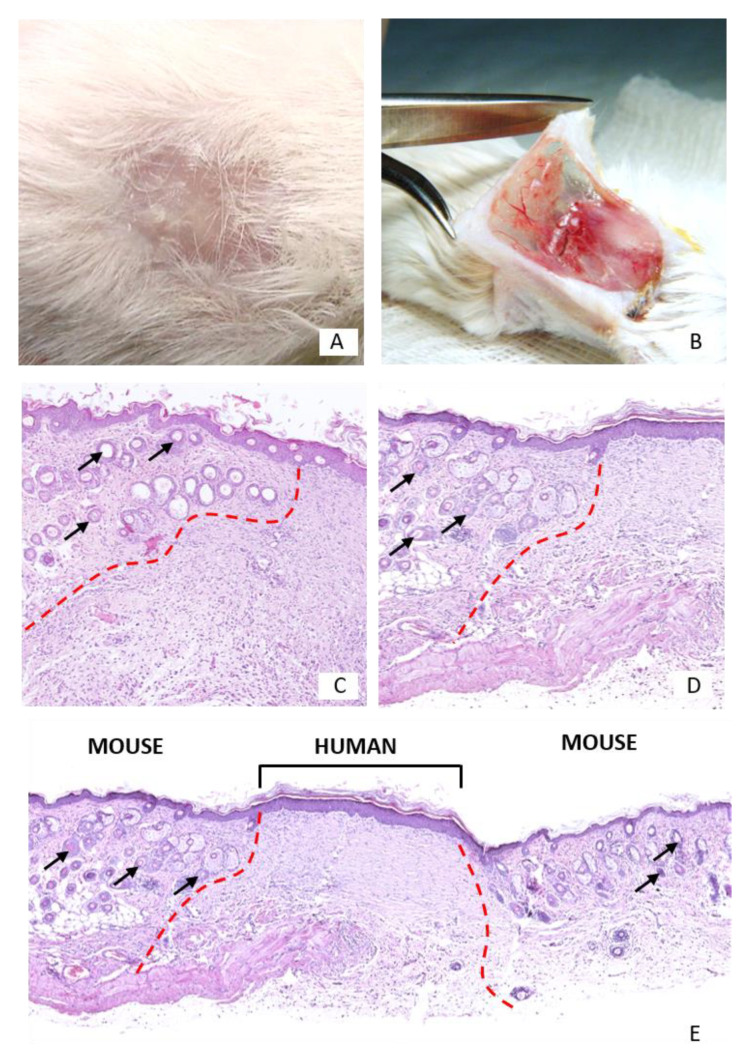
(**A**,**B**)—Keratinocytes preserved for 3 months and transplanted for 21 days. (**A**)—A completely healed wound, (**B**)—Full blood supply to the graft bed. (**C**–**E**)—Histologic picture of newly formed epidermis originated from transplanted human keratinocytes isolated from fresh skin (**C**) and skin preserved in anhydrous NaCl for 3 months (**D**,**E**). The border between the human and mouse epidermis is marked with a red line. The human dermis lacks the hair follicles characteristic of the mouse dermis (arrows). Slides stained with hematoxylin-eosin, magnification 10× (**C**,**D**) and 5× (**E**).

After 21 days of follow-up, complete wound healing was observed, and a full blood supply to the graft bed was visible. In the histology, no differences in healing were observed between control (fresh KCs) and experimental (preserved KCs) groups (Figure 5).

### 3.5. Antigen Expression in the Newly Formed Epidermis Depending on the Time of Skin Preservation

#### 3.5.1. HLA Expression

**Figure 6:**
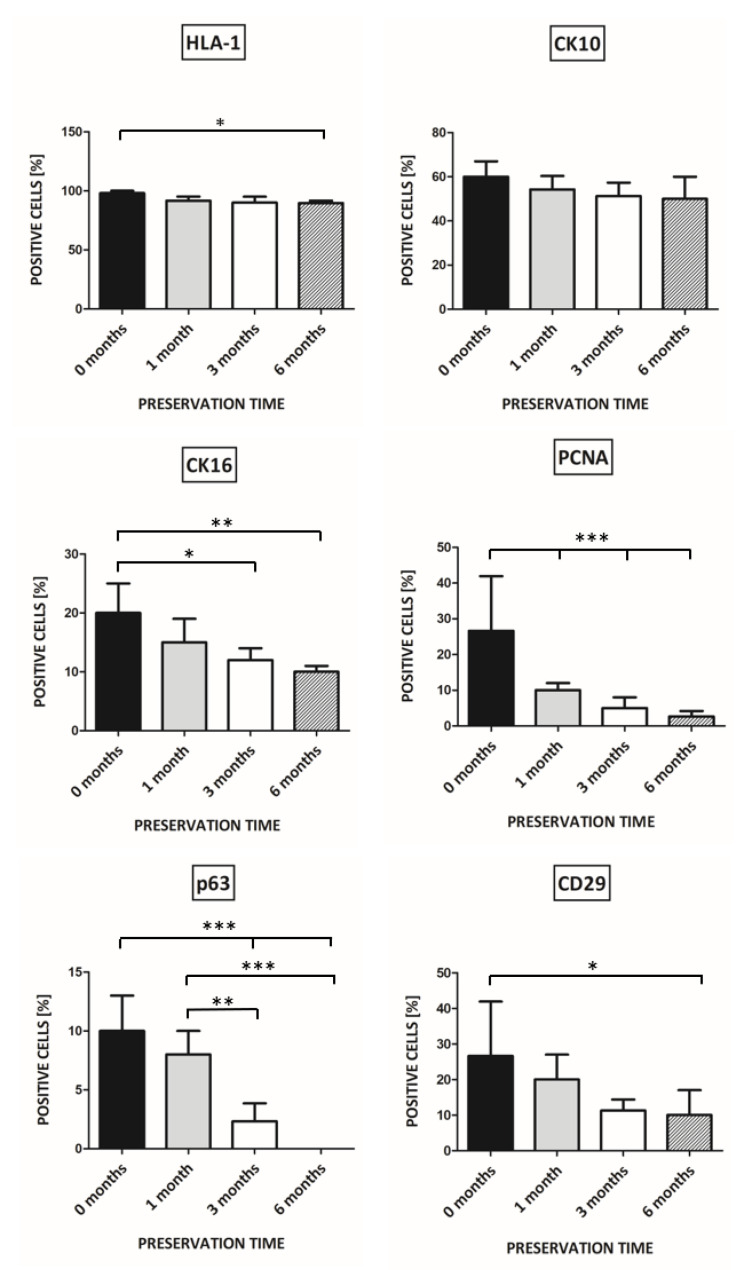
The percentage of keratinocytes in newly formed epidermis positive for HLA-1, CK10, CK16, PCNA, p63, and CD29 depending on the time of preservation. Statistically significant results are shown at the bar graph (* *p* < 0.05, ** *p* < 0.01, *** *p* < 0.001).

The percentage of KCs positive for HLA-1 isolated from skin preserved in anhydrous NaCl for the time ranging from 1 to 6 months was approximately 10% lower than in the non-preserved control (Figure 6). After 6 months of preservation, the difference was statistically significant (*p* < 0.05). An analysis was performed using one-way ANOVA followed by Tukey’s Multiple Comparison Test (F_3,16_ = 4.459, *p* = 0.0185).

#### 3.5.2. CK10 and CK16 Expression

The percentage of KCs positive for CK10 isolated from skins preserved in anhydrous NaCl was approximately 50% for the preservation time ranging from 1 to 6 months, in comparison to 60% ± 7.0 for non-preserved control (Figure 6). ANOVA did not reveal statistical significance between the studied groups.

The decrease in the percentage of KCs positive for CK16 was statistically significant regarding KCs isolated from skins preserved for 3 (*p* < 0.05) and 6 months (*p* < 0.01) in comparison to 20.0 ± 5.0 for control (F_3,16_ = 7.721, *p* = 0.0021), (Figure 6).

#### 3.5.3. PCNA, p63, and CD29 Expression

The percentage of KCs positive for PCNA isolated from skin preserved in anhydrous NaCl for a time from 1 to 6 months was statistically significantly lower in comparison to controls (*p* < 0.001) and ranged from 26.7 ± 15.3 (control) to 2.7 ± 1.5 (6 months) (F_3,16_ = 23.55, *p* < 0.0001) (Figure 6).

The percentage of cells expressing p63 was 10 ± 3.0% in the control group. With the length of skin preservation, we observed a decrease in p63 positive cells to 0% after 6 months (Figure 6). The results were statistically significant for 0 vs. 3 months, 0 vs. 6 months, and 1 vs. 6 months (*p* < 0.001), as well as for 1 vs. 3 months (*p* < 0.01) (F_3,16_ = 28.73, *p* < 0.0001).

CD 29 expression was significantly lower when comparing keratinocytes from non-preserved skin to skin preserved for 6 months (*p* < 0.05) (F_3,16_ = 3.541, *p* = 0.0387). (Figure 6).

#### 3.5.4. Expression of CD1a, CD4, CD8, CD20

No human Langerhans cells or skin T and B lymphocytes were found in the grafts.

**Figure 7:**
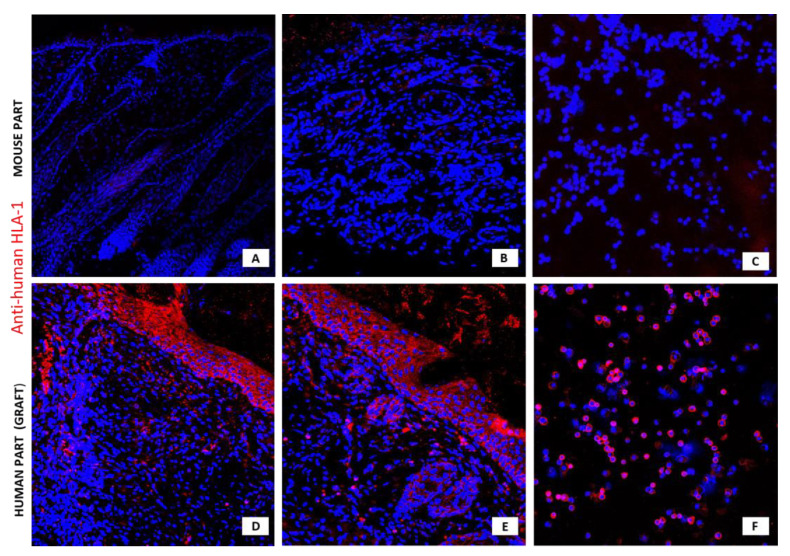
Confocal microscopy images of specimens stained with anti-human HLA-1 Ab. Mouse part of the specimen (**A**,**B**) and the newly formed epidermis after transplantation of human KCs into the wound (**D**,**E**). Cells isolated from the mouse skin (**C**) and from the newly formed epidermis in the graft area (**F**). Grafted keratinocytes were isolated from skin preserved in anhydrous NaCl for 1 month. Red-stained cells expressing HLA-1 (Alexa Fluor 633). Nuclei stained blue (Hoechst), magnification 5× (**A**) and 10× (**B**–**F**).

**Figure 8:**
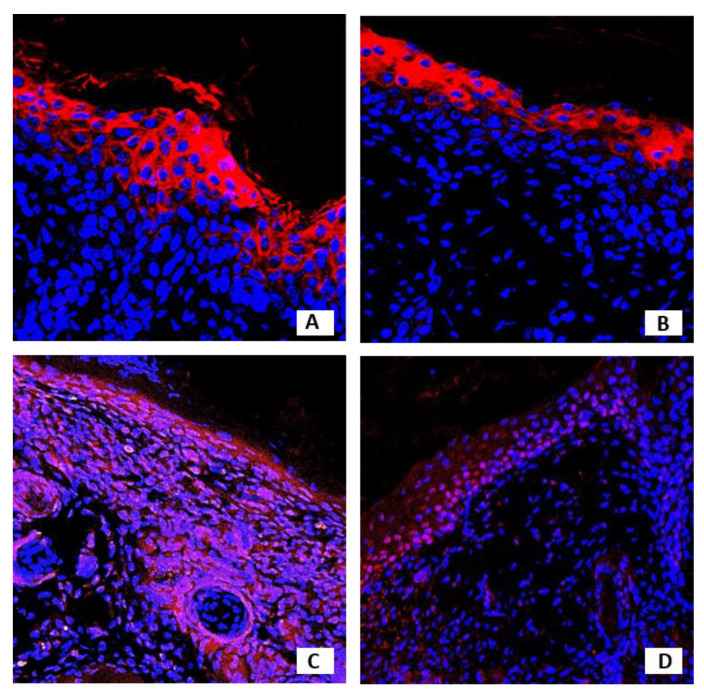
Confocal microscopy images of the newly formed epidermis. Grafted keratinocytes were isolated from skin preserved in anhydrous NaCl for 1 month. (**A**) Red-stained cells expressing CK10 (Alexa Fluor 633), nuclei stained blue (Hoechst), magnification 40×. (**B**) CK 16 staining, magnification 40×. (**C**) PCNA staining, magnification 20×. (**D**) p63 staining, magnification 40×.

Confocal microscopy images of investigated antigens expression in the newly formed epidermis are shown in Figure 7 and Figure 8.

### 3.6. Metabolic Activity of Keratinocytes Isolated from Skin Preserved in Anhydrous NaCl

The metabolic activity of KCs isolated from: fresh skin, skin preserved for 1 month, and skin preserved for 1 month and transplanted for 21 days was measured (Table 3). The activities of most enzymes were comparable between the above groups. The activity of valine arylamidase and cystine arylamidase, in keratinocytes isolated from the newly formed epidermis, was lower than in the other two groups. For α-galactosidase, β-glucosidase, and α-fucosidase, a higher enzymatic activity was observed in the group of KCs originating from the newly formed epidermis, in comparison to the others.

## 4. Discussion

There is a great demand to improve efficient methods of KCs preservation for transplantation. Autologous and allogeneic frozen keratinocytes stored in liquid nitrogen are used to enhance wound healing [13,19,20]. Most cryopreservation methods involve fetal calf serum (FCS) and/or dimethylsulfoxide (DMSO). Although these methods are efficient, DMSO exhibits cell toxicity in vitro and in vivo. At high concentrations, this can cause the disintegration of the lipid bilayer [21]. There are also studies on applying HES (Hydroxyethyl starch) in the cryopreservation of keratinocytes [22]. The addition of HES was found to increase the rate of viability after freezing [22]. However, the high costs, the need for qualified staff, and the appropriate cryopreservation equipment are not always achievable in low- and middle-income countries. Our experimental model presented in this manuscript may be an alternative to KCs storage in liquid nitrogen. The integration and survival of cells are crucial factors following transplantation. Therefore, we investigated the viability, antigen expression, and metabolic activity of anhydrous NaCl-preserved and grafted keratinocytes. The mechanism of preserving the molecular structure and function of cells by sodium chloride was not described in the literature. It might be a sodium-specific, chloride anion, and hyperosmolarity effect. Hypertonic-inhibited N-formyl-methionyl-leucyl-phenylalanine (fMLP) stimulated an increase in intracellular calcium and shedding of receptors [23]. The osmotic dehydration of cells and intercellular matrix should also be taken into consideration. Sodium chloride penetrated the whole skin specimen and did not crystallize. Crystals were not seen under the operating microscopes (magnification 40×) on cross-sections of the midportions of specimens.

### 4.1. Immunogenicity of Keratinocytes Isolated from Anhydrous NaCl-Preserved Skin

Allogeneic skin transplantation is associated with an immune response leading to donor cell elimination and graft rejection.

After skin transplantation, dendritic cells (DCs) migrate from the graft via lymphatic vessels and infiltrate the recipient’s draining lymph nodes. There, they present donor antigens via two mechanisms: a direct pathway in which T cells detect donor MHC antigens on donor DCs, and an indirect pathway in which T cells recognize donor peptides linked to MHC molecules on recipient DCs [24].

In our previous studies, we discovered that the preservation of some tissues, e.g., aortas in anhydrous NaCl, reduces their immunogenicity [8].

On the other hand, experiments on skin preserved in anhydrous NaCl showed that the expression of antigens such as CD1a (Langerhans cells), CD 2,3,4 (T lymphocytes) CD14 (monocytes), CD15 (granulocytes), CD22 (B cell), CD68 (macrophages) and HLA-DR (MHC class II) was maintained [6]. As above, antigens are characteristic of immune cells and involved in immune reaction/rejection; their high expression in the skin for grafting is not desirable.

The transplantation of isolated keratinocytes, instead of whole skin, would not only lower host response but also allow graft versus host disease (GVHD) to be avoided. The most common clinical manifestation of GVHD relates to the skin, as the epidermis is equipped with immune-competent cells. We proved that, both among the transplanted population of KCs and cells isolated from the newly formed epidermis, there are no T cells. This cell population is also free of Langerhans and B cells.

### 4.2. Viability of Keratinocytes Isolated from Anhydrous NaCl-Preserved Skin

Viable skin allografts are considered by many practitioners to show a better outcome in promoting neovascularization, speeding up healing, and stimulating immunomodulatory response than nonviable [10,11]. A higher viability is considered to be associated with better engraftment and an improvement of tissue granulation [10,25]. However, grafts of reduced viability may act as a temporary dressing causing the promotion of re-epithelialization of the wound. High viability may not be essential for skin allograft function [10,25]. Another issue is that all methods of skin preservation result in a decrease in viability.

The most commonly used procedure is cryopreservation; glycerol preservation and storage at +4 °C are less commonly used [11]. The viability of fresh skin decreases quickly during storage at +4 °C. The viability percentage was 56% at day 2 and 42% at day 7 for Ringer Lactate, whereas skin retained a viability of 87% at day 2 and 60% at day 7 when stored in RPMI [11]. Cryopreservation maintains some viability, despite the cellular trauma of freezing and thawing. Most authors agree that acceptable viability is obtained by a storage temperature of −80 °C or below. Glycerol-preserved allografts are not viable [10,11]. Udoh et al. reported that the cell survival rates of the grafts cryopreserved at −135 °C for 1 month, 6 months, and 1 year were 89.3%, 61.7%, and 61.6%, respectively [26]. Cryopreservation at −80 °C maintained cell viability for 1 month, but after 6 months of cryopreservation, viability decreased to 35.2% [26]. In different studies, authors showed a reduction in cell viability of 50% as soon as after 2 weeks at −80 °C [10]. The loss of cell viability in this study was 65% and 69% after 6 and 12 months, respectively, compared with fresh skin [10]. Schiozer et al. estimated the viability of cultured epithelium after storage at −70 °C. The viability of cells reached 37%, 25%, and 15% of the control values after 1, 6, and 12 months, respectively [27].

Our studies showed that the viability of KCs isolated from skin preserved in anhydrous NaCl decreased with preservation time. The viability of isolated KCs remained stable within the first 3 weeks of preservation and decreased only by 10%. A further decrease was observed following one further week (to a viability of 68.3% ± 10.4). After 3 months and 6 months of storage, only 26.7% ± 12.6 and 18.3% ± 6.5 KCs, respectively, remained viable.

Considering viability, cryopreservation at temperatures below −80 °C provided a significantly better outcome compared to preservation in anhydrous NaCl. On the other hand, methods such as preservation at −70 °C, glycerol preservation, and storage at +4 °C are not comparable in this aspect.

### 4.3. Morphology of Keratinocytes Isolated from Anhydrous NaCl-Preserved Skin

Skin preservation in anhydrous NaCl did not affect the morphology of KCs stored up to 3 months. MGG staining and cell culture observation revealed that keratinocytes of all layers maintained their morphology.

If we consider cryopreservation, in a histological examination, the cell structure and basal layer were very well preserved after 6 months of storage at −135 °C after slow freezing. At temperatures higher than this, ice crystals form, and recrystallization occurs in the cells, causing cellular lesions [26].

It is well known that glycerol-preserved keratinocytes do not retain their viability. Despite this, glycerol treatment does not affect the fundamental structural integrity of the skin. When comparing skin preserved in 85 percent glycerol to untreated skin, large parts of treated skin were well-preserved. In some parts of the epidermis, cells were shrunken, but the dermal part of the glycerol-treated skin was morphologically unaltered [28].

The results for KCs preserved for a long time in NaCl are similar to those of KCs preserved in glycerol. After preservation in anhydrous NaCl lasting over 3 months, a severe decrease in viability was observed. At the same time, KCs retained their morphology and function in wound healing.

### 4.4. The Optimal Number of KCs for Grafting

In our study design, the expression of investigated antigens rose with the number of transplanted cells up to 7 × 10^6^ KCs grafted. In the case of 7 × 10^6^ and 14.0 x10^6^ transplanted KCs, the expression of antigens was comparable. The clinical outcome in terms of the epithelialization area of the wound bed stood in agreement with histology. We did not observe further significant clinical improvement in the wound healing for KCs transplanted at concentration over 7 × 10^6^ per standard wound of 1.5 cm in diameter.

In the research conducted by other authors, the number range of transplanted keratinocytes in clinical settings varied depending on the type of the wound. With a single administration, this usually ranges from 3 × 10^6^/mL or 6 × 10^6^/mL [29] up to 10 × 10^6^ [30].

Lichti et al. proved that immunodeficient mice injected with 5–8 × 10^6^ primary keratinocytes, plus 5 × 10^6^ primary fibroblasts, reconstituted normal haired skin in the transplant site [18].

Wounds can also be treated with keratinocytes at a rate of 0.5×10^6^, administered every 14 days [31].

### 4.5. Follow-Up and Endpoint of Wound Healing

In the studies of Yanaga et al. on cryopreserved, cultured epidermal allografts, the authors proved that the sex-determining region Y (SRY) gene could be detected only 2–4 weeks after cell transplantation [32]. This is evidence that cryopreserved cultured epidermal allografts are temporally but not permanently taken by the recipient.

The above results are consistent with our observation that, after 30 days, the expression of human antigens in the transplant in SCID mice decreases, and mice cells populate the graft. On the other hand, in our experimental design, the 7 days of follow-up turned out to be too short for the complete formation of the human epidermis.

### 4.6. Antigen Expression in the Newly Formed Epidermis Depending on the Time of Skin Preservation

Previously, an attempt was made to transplant keratinocytes obtained from skin preserved for 12 months [5]. From this pilot study, we concluded that, after such a long-term preservation time, only cells from *stratum granulosum* could survive. Almost all obtained KC did not express p63, and CD29 were assumed to be markers specific for stem cells, and they did not proliferate. This is why we decided to perform further and more detailed studies on cells isolated from skin preserved for up to 6 months.

The expression of HLA-1 and CK10 remains unchanged after a preservation of up to 6 months. After 3 months of preservation, we observed a rapid decrease in cells expressing p63 and CD29 in the newly formed epidermis. The percentage of KCs positive for PCNA decreased to around one-third of the control value as soon as 1 month after preservation. After 6 months of preservation, only 10% of KCs isolated from the newly formed epidermis showed the expression of a PCNA marker, which determined the regenerative potential of transplanted cells. These results are consistent with the data obtained from experiments on viability. Thus, we believe that the optimal storage time would be up to 3 months.

Having a large number of viable keratinocytes available (e.g., after preservation in anhydrous NaCl for less than one month) makes it possible to transplant cells immediately after isolation. If the number of viable cells is insufficient (e.g., after 3 or more months of preservation), cell culture may be an option, as our studies showed that viable cells obtained from skin preserved in NaCl retain their proliferative potential.

### 4.7. Metabolic Activity of Keratinocytes Isolated from Skin Preserved in Anhydrous NaCl

API ZYM test was designed to investigate enzymatic activities and provide semiquantitative information on hydrolytic enzymes’ activity associated with the biodegradation of lipids, proteins, carbohydrates, and nucleic acids. Although the API ZYM assay is especially dedicated to bacteria, this technique, according to the manufacturer’s description, applies to all specimens (microorganisms, cell suspensions, tissues, etc.). The activity of most enzymes was comparable between fresh, preserved, and preserved and subsequently transplanted KCs, providing evidence that storage in anhydrous NaCl maintains the metabolic functions of skin cells. In the case of valine arylamidase and cystine arylamidase, their activity in keratinocytes isolated from the newly formed epidermis was lower than in the other two groups. It is possible that anhydrous NaCl impaired enzyme synthesis, and cells after preservation and transplantation revealed only a partial function of their metabolic activity. On the other hand, for α-galactosidase, β-glucosidase, and α-fucosidase, a higher enzymatic activity was observed in the group of KCs originating from the newly formed epidermis in comparison to the others. A possible explanation for this phenomenon is that the graft may contain the low addition of mouse cells. Mouse cells may have migrated to the wound bed and exhibited higher enzymatic activity than human cells, resulting in this outcome. However, it is not clear why an increase in activity was only observed for α-galactosidase, β-glucosidase, and α-fucosidase.

### 4.8. A Shortcoming of the Studies

All results described in this paper were obtained in severe combined immunodeficiency (SCID) mice lacking both T and B lymphocytes and accepting xenogeneic cells. This model provided us with the opportunity to evaluate the therapeutic potential of keratinocytes isolated from skin preserved in anhydrous NaCl, without the necessity of considering the influence of the host immune system response on the grafted cells. In the above studies, we managed to establish the optimal conditions regarding the transplantation of KCs obtained from skin preserved in anhydrous NaCl. At this stage, we proved that no immune passenger cells are transplanted, with a fraction of KCs isolated from skin preserved in anhydrous NaCl, and that this method is less immunogenic than transplanting whole skin. The next step is to perform transplants in animals without immunodeficiency. Experiments on syngeneic and allogeneic rats that mimic the clinical situation of auto- and allografts in humans are planned.

## 5. Conclusions

We developed a method for the long-term preservation of human skin for isolation and effective KCs transplantation to wounds. KCs isolated from human skin preserved in anhydrous NaCl at 4 °C for months can be successfully transplanted to SCID mice.

The originality of this method consists of an effective storage procedure and an easy method of KCs preparation for transplantation. The preservation in anhydrous, pulverized NaCl inhibits KCs proliferation but did not stop some cells from displaying enzymatic activity upon setting in culture. Transplantation to SCID mice brought about restarting of mitoses. Our method has practical implications. KCs transplanted to the wound provide a physiological barrier. They may also be a source of proliferating transient cells and cytokines regulating the growth of their own and host keratinocytes and fibroblasts. Deep frozen tissues do not meet these conditions because their cellular structure can be destroyed during thawing. Further tests on animals with fully functional immune systems are needed. This research will aim to identify KCs providing the signal for a high proliferative capacity.

## Figures and Tables

**Table 1:** Viability of keratinocytes isolated from anhydrous NaCl-preserved skin.

	0d (KCs from Fresh Skin)	7d	14d	21d	1m	3m	4m	5m	6m	7m	12m
Viability (%)	94.0 ± 4.6	90.7 ± 5.1	85.0 ± 5.0	85.0 ± 13.2	68.3 ± 10.4	26.7 ± 12.6	21.3 ± 6.1	18.3 ± 4.7	18.3 ± 6.5	12.7 ± 6.8	11.7 ± 2.9

**Table 2:** Experimental groups—keratinocytes isolated from fresh or preserved skin and transplanted.

Preservation Time	Follow-Up Time	Number of Transplanted Keratinocytes [×10^6^]	Number of Grafts
0 months (KCs isolated from fresh skin)	21 days	7	5
1 months	21 days	7	5
3 months	21 days	7	5
6 months	21 days	7	5

**Table 3:** Metabolic activity of keratinocytes.

	Enzyme	KCs Isolated from Fresh Skin	KCs Isolated from Skin Preserved for 1 Month	KCs Isolated from Skin Preserved for 1 Month and Transplanted for 21 Days
1	Control	0	0	0
2	Alkaline phosphatase	5	5	5
3	Esterase (C4)	3	3	3
4	Esterase lipase (C8)	3	3	3
5	Lipase (C14)	0	0	0
6	Leucine arylamidase	5	5	5
7	Valine arylamidase	5	5	3
8	Cystine arylamidase	1	1	0
9	Trypsin	3	3	3
10	α-chymotrypsin	0	0	0
11	Acid phosphatase	5	5	5
12	Naphtol AS-BI -phosphohydrolase	5	5	5
13	α-Galactosidase	3	0	5
14	β-Galactosidase	5	4	5
15	β-Glucuronidase	5	5	5
16	α-Glucosidase	1	1	0
17	β-Glucosidase	1	2	3
18	N-acetyl-β-glucosaminidase	5	4	3
19	α-Mannosidase	0	0	1
20	α-Fucosidase	1	1	3

5 ≥ 40 nM**,** 4 = 30 nM**,** 3 = 20 nM**,** 2 = 10 nM**,** 1 = 5 nM.

## Data Availability

The data that support the findings of this study are available on request from the corresponding author.
